# Multiplex single-nucleotide polymorphism typing of the human Y chromosome using TaqMan probes

**DOI:** 10.1186/2041-2223-2-13

**Published:** 2011-05-31

**Authors:** Begoña Martínez-Cruz, Janet Ziegle, Paula Sanz, Graciela Sotelo, Roger Anglada, Stéphanie Plaza, David Comas

**Affiliations:** 1 Institut de Biologia Evolutiva (CSIC-UPF), Departament de Ciències Experimentals i de la Salut, Universitat Pompeu Fabra, Doctor Aiguader 88, E-08003 Barcelona, Spain; 2 Applied Biosystems, Inc., 850 Lincoln Centre Drive, Foster City, CA 94404, USA; 3 Genome Facility Core Service, Universitat Pompeu Fabra (UPF), Doctor Aiguader 88, E-08003 Barcelona, Spain

## Abstract

**Background:**

The analysis of human Y-chromosome variation in the context of population genetics and forensics requires the genotyping of dozens to hundreds of selected single-nucleotide polymorphisms (SNPs). In the present study, we developed a 121-plex (121 SNPs in a single array) TaqMan array capable of distinguishing most haplogroups and subhaplogroups on the Y-chromosome human phylogeny in Europe.

**Results:**

We present data from 264 samples from several European areas and ethnic groups. The array developed in this study shows >99% accuracy of assignation to the Y human phylogeny (with an average call rate of genotypes >96%).

**Conclusions:**

We have created and evaluated a robust and accurate Y-chromosome multiplex which minimises the possible errors due to mixup when typing the same sample in several independent reactions.

## Background

The development of high-throughput technologies to genotype hundreds of thousands of markers has yielded an increase in the understanding of the genetic diversity of our species [1]. This genomic knowledge has been applied to different fields, from biomedical and pharmaceutical research to population genetics and forensics [2-5]. Most of the genotyping technologies have been based on typing of single-nucleotide polymorphisms (SNPs) that commonly have only two alleles (ancestral or derived compared to nonhuman primates) and have usually arisen once. These high-throughput SNP genotyping analyses provide much information about the variation of our genome, but still little information has been derived from some specific genome regions of great interest. SNPs are also used for human identification purposes and to reconstruct human demographic history, although these fields require the typing of a few selected SNPs for targeted research rather than the use of high-throughput genotyping methods (that is, allele-specific probes or single base primer extensions).

The human Y chromosome has been extensively analysed in forensic and evolutionary studies because of its unique properties. Despite being a complex chromosome with highly repetitive sequences [6], its exclusively paternal inheritance due to the lack of recombination over most of the chromosome has allowed researchers to trace paternal lineages and reconstruct the male demographic history of populations [7]. The human Y chromosome contains hundreds of well-characterised SNPs (around 600) whose evolutionary relationships have been established in a robust phylogeny [8]. The Y-chromosome phylogeny defines several main branches (haplogroups) named with alphabetical (A to T) and numerical codes. Each branch is subdivided into more minor braches (subhaplogroups) defined by other SNPs in a hierarchical way [9]. The classification of a male DNA sample within the Y-chromosome phylogeny requires the successive typing of markers that define the haplogroup and subhaplogroups to which the sample belongs. This process is not only laborious but also time-consuming. For this reason, several attempts to genotype multiple Y-chromosome SNPs in a single reaction have been reported, mainly using single-base extension methodologies [10-15] or oligonucleotide ligation assays [16]. Nonetheless, these multiplex approaches have not been able to type successfully more than 35 Y-chromosome SNPs in a single reaction.

It is preferable to genotype, in a reproducible manner, around 100 SNPs at a time. We therefore aimed to design a single multiplex reaction of this size using TaqMan probes (Applied Biosystems, Inc., Foster City, CA, USA). TaqMan probes are robust and reproducible assays that have been used extensively to type SNPs in single reactions, that is, one SNP per TaqMan reaction. Each assay is based on TaqMan probes that are hydrolysis probes that anneal within a specific DNA region amplified by polymerase chain reactions (PCRs). The probes present a fluorophore (usually VIC or 6-FAM, Applied Biosystems) attached to the 5′-end and a quencher at the 3′-end that prevents fluorescence of the fluorophore. As the AmpliTaq Gold DNA Polymerase (Applied Biosystems) extends the primer, its exonuclease activity degrades the probe that has annealed to the template and releases the fluorophore, relieving the quenching effect and allowing fluorescence of the fluorophore. In a TaqMan assay, two probes labelled with different fluorophores are used, each one complementary to one of the two alleles (ancestral or derived) of a SNP. In this way, in a real-time PCR, the alleles of one SNP of a DNA sample can be interrogated using two TaqMan probes labelled with different fluorophores. In the present study, our goal was to perform more than 100 TaqMan assays in a single array to define detailed haplogroups and subhaplogroups of the human Y chromosome in European populations.

## Results

**Figure 1:**
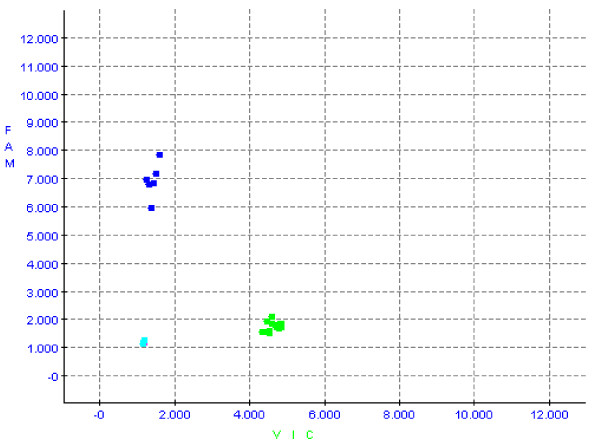
**Autocaller plot showing the fluorescence of 22 samples (green and dark blue) and two nontemplate controls (light blue) in the M203 assay**. The TaqMan probe complementary to the ancestral allele (G) was labelled with the VIC fluorophore, whereas FAM was used to label the derived allele (C). Three clusters are seen in the plot: the samples with the derived allele, thus belonging to the Y-chromosome haplogroup branch DE, are shown in dark blue; the samples with the ancestral allele, thus belonging to any haplogroup except DE, are shown in green; and the negative controls are shown in light blue.

Of the 128 SNPs designed in the TaqMan OpenArray plate (Applied Biosystems, Inc.), 121 were successfully typed. The internal controls gave completely concordant results, as M145 genotypes were always concordant with M203, and the two different assays for the marker M9 gave identical results in all the individuals. In addition, the genotypes obtained for the control samples (Coriell samples; Coriell Institute for Medical Research, Camden, NJ 08103 USA) were identical to those previously obtained with the same TaqMan assays used one-by-one, giving a concordance rate of 100% (Table 1). An example of a successful assay for the M203 marker is shown in Figure 1. Three clusters of samples are shown in the plot from the Autocaller software (Applied Biosystems): those with VIC fluorescence (representing the ancestral allele, G, of the M203 marker); those with FAM fluorescence (representing the derived allele, C); and the negative controls, nontemplate controls (NTCs) without VIC or FAM fluorescence.

**Table 1:** Genetic markers analysed in the OpenArray, the Y haplogroup they define, the alleles and their call rates (successful genotype calls)

List of genetic markers analysed				List of genetic markers analysed (continuation)				List of genetic markers analysed (continuation)			
		
Marker	Haplogroup^a^	Alleles^b^	Call rate (%)	Marker	Haplogroup^a^	Alleles^b^	Call rate (%)	Marker	Haplogroup^a^	Alleles^b^	Call rate (%)
M216	C	C/T	95.8	P259	I1d	T/G	96.6	M231	N	G/A	94.7
M168	CT	C/T	98.1	P215	I2	A/G	95.1	M128	N1a	2-bp del	97.0
M174	D	T/C	97.7	P37.2	I2a	T/C	96.6	P43	N1b	G/A	96.6
M145^c^	DE	G/A	96.6	P41.2	I2a1	T/C	97.3	P105	N1c	G/A	96.2
M203^c^	DE	G/C	96.6	M26	I2a2	G/A	95.5	M214	NO	G/C	96.6
M96	E	G/C	98.1	M223	I2b	C/T	96.2	M119	O1a	A/C	99.2
P147	E1	T/A	97.7	M284	I2b1	ACAA/del	96.2	P31	O2	T/C	95.8
M33	E1a	A/C	95.5	M379	I2b2	GT/del	97.0	M122	O3	T/C	98.9
P177	E1b	C/T	93.6	P95	I2b4	G/T	95.1	M45	P	G/A	96.2
P2	E1b1	C/T	97.3	P123	IJ	T/C	94.3	M242	Q	C/T	97.3
M2	E1b1a	A/G	97.0	M304	J	A/C	94.3	M207	R	A/G	95.5
M215	E1b1b	A/G	97.3	M267	J1	T/G	95.5	M173	R1	A/C	93.2
M35	E1b1b1	G/C	95.8	M62	J1a	T/C	95.5	SRY10831.2	R1a	G/A	99.6
M78	E1b1b1a	C/T	98.1	M365	J1b	A/G	Failed	M56	R1a1a	A/T	97.7
V12	E1b1b1a1	A/G	97.3	M390	J1c	A/ins	Failed	M157	R1a1b	A/C	98.1
V13	E1b1b1a2	G/A	97.0	P56	J1d	A/G	96.6	M204	R1a1c	T/G	95.8
V22	E1b1b1a3	T/C	97.7	P58	J1e	T/C	95.5	P98	R1a1d	C/T	Failed
V65	E1b1b1a4	G/T	96.2	M172	J2	T/G	96.6	PK5	R1a1e	C/T	95.8
M81	E1b1b1b	C/T	89.8	M410	J2a	A/G	95.5	M343	R1b	C/A	97.3
M123	E1b1b1c	G/A	97.7	M47	J2a1	G/A	96.6	M18	R1b1a	2-bp ins	96.6
M281	E1b1b1d	G/A	97.0	M340	J2a10	G/C	94.7	P297	R1b1b	G/C	95.8
V6	E1b1b1e	G/C	99.6	M419	J2a11	AAAAG/del	95.8	M73	R1b1b1	2-bp del	96.2
P72	E1b1b1f	G/A	98.1	P81	J2a12	C/T	Failed	M269	R1b1b2	C/T	96.2
M329	E1b1c	G/C	98.1	P279	J2a13	G/A	98.1	L23	R1b1b2a	A/G^e^	84.8
P75	E1b2	G/A	Failed	M67	J2a2	A/T	95.1	P311	R1b1b2a1	A/G	97.0
M75	E2	G/A	97.7	M92	J2a2a	T/C	96.2	U106	R1b1b2a1a	C/T	97.3
M89	F	C/T	97.7	M68	J2a3	A/G	91.3	U198	R1b1b2a1a1	G/A	98.5
M201	G	G/T	95.5	M137	J2a4	T/C	97.7	P107	R1b1b2a1a2	G/A	Failed
M285	G1	G/C	96.6	M158	J2a5	G/A	97.7	P312	R1b1b2a1b	A/C^e^	97.3
P20	G1a	C/del	95.8	M289	J2a6	G/A	96.6	M65	R1b1b2a1b1	A/T	96.6
P76	G1b	G/C	98.9	M318	J2a7	T/C	97.3	M153	R1b1b2a1b2	T/A	97.7
P287	G2	G/T	96.6	M319	J2a8	T/A	97.7	SRY2627	R1b1b2a1b3	C/T	97.3
P15	G2a	C/T	97.7	M339	J2a9	T/G	97.7	U152	R1b1b2a1b4	C/T	97.0
M287	G2b	A/T	97.0	M12	J2b	G/T	97.0	M126	R1b1b2a1b4a	4-bp del	97.3
M377	G2c	A/G	94.7	M205	J2b1	T/A	98.1	M160	R1b1b2a1b4b	A/C	96.2
M69	H	T/C	97.0	M241	J2b2	G/A	Failed	L21	R1b1b2a1b5	C/G	97.7
M52	H1	A/C	95.5	M9a^d^	K	C/G	97.3	M37	R1b1b2a1b5a	C/T	87.9
Apt	H2	G/A	92.8	M9b^d^	K	C/G	97.3	M222	R1b1b2a1b5b	G/A	97.7
M170	I	A/C	97.0	M20	L	A/G	94.3	P66	R1b1b2a1b5c	G/A	98.1
M253	I1	C/T	97.0	M27	L1	C/G	94.7	M335	R1b1c	T/A	96.2
M21	I1a	A/T	96.2	M317	L2	GA/del	94.3	M124	R2	C/T	98.1
M72	I1b1	A/G	98.1	M357	L3	C/A	97.3	M70	T	A/C	97.0
P109	I1c	G/A	95.8	P256	M	G/A	97.7				

^a^Haplogroups are based upon data published by Karafet *et al*. [8] and on the Internal Society of Genetic Genealogy (ISOGG) 2009 Y-DNA SNP Index website (http://isogg.org/tree/ISOGG_YDNA_SNP_Index09.html).

^b^The two alleles of each marker (ancestral or derived) are shown.

^c^Both markers (M145 and M203) define the same branch of the human Y-chromosome phylogeny (haplogroup DE).

^d^Marker M9 was genotyped with two independent assays, with TaqMan probes labelled with different fluorophores (see "SNP selection" inside the Material and Methods section).

^e^Ancestral allele not available.

**Figure 2:**
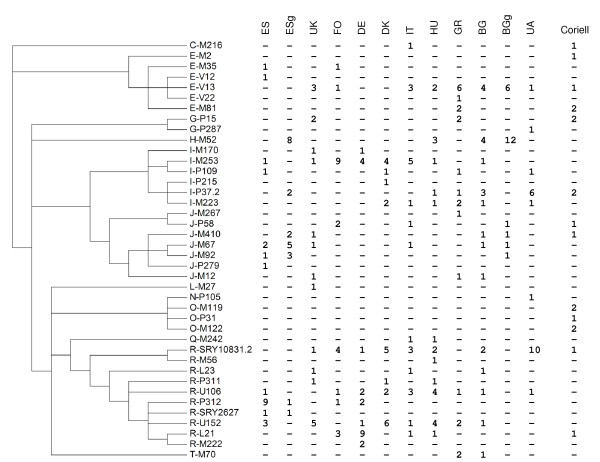
**Phylogenetic tree showing the haplogroup counts found in the present sample set following the nomenclature published by Karafet *et al***. [8] and on the Internal Society of Genetic Genealogy (ISOGG) 2009 Y-DNA SNP Index website (http://isogg.org/tree/ISOGG_YDNA_SNP_Index09.html). ES, Spanish; ESg, Spanish Roma; UK, British; FO, Faroe Islands; DE, German; DK, Danish; IT, Italian; HU, Hungarian; GR, Greek; BG, Bulgarian; BGg, Bulgarian Roma; UA, Ukrainian.

The number of successful genotypes for the remaining 121 SNPs in the pool (the call rate) was high (average 96.4%), with the lowest value being for L23 (84.8%) (Table 1). The combination of 121 SNPs was able to distinguish a total of 118 different haplogroups and subhaplogroups (Additional file 1, Figure S1), 40 of which were present in the populations typed and 24 of which were shared by two or more European samples (Figure 2). Haplogroup composition varied greatly across the different populations sampled, as expected from the known phylogeography of the samples used.

Of 282 individuals where genotyping was attempted, three did not yield any amplified product for any assay. This failure was due to technical manipulation problems in the DNA loading phase, as the wells corresponding to these three samples were empty in the array. Of the remaining 279, 277 showed complete phylogenetic compatibility. If a sample showed the derived allele for a SNP that led to a certain branch of the tree, it also presented the derived alleles for all the SNPs leading to that branch and the ancestral alleles for the SNPs leading to other branches. For example, if a sample was derived for M201 (allele T), it was also derived for M89 (allele T), M168 (allele T), M42 (allele T) and SRY_10831.1 _(allele G), but was ancestral for all the rest of the SNPs in the array (see Table 1). Although some of the haplogroups (that is, haplogroups D and M) were not present in the populations sampled, all the samples typed showed the ancestral allele for the SNPs defining these branches. Two individuals showed phylogenetic incompatibilities; that is, we found derived alleles for SNPs leading to different branches of the phylogeny in the same individual. In both cases, this incongruence was due to a single misassigned internal SNP (false-positive) that was eliminated, and both individuals could be correctly assigned in the phylogeny. However, it should be noted that if the misassignation occurs at the very end of the branch where the sample is assigned in the phylogeny, it might be undetectable and would lead to the wrong assignation at the subbranch level.

## Discussion

The aim of the present study was to create a SNP multiplex for the human Y chromosome in a single array that could be used to define Y-chromosome haplogroups in European populations. Previously, genotyping of Y-chromosome SNPs has been performed in single-plex reactions or using limited multiplexes [10-16]. The multiplex SNP typing described in the present study greatly reduces the amount of time spent on typing. Besides that, typing a large number of SNPs in a single array avoids the possibility of errors by sample mixup when typing the same sample in several independent reactions. The pass rate (>94% successfully genotyped SNPs) and the average call rate (>96% successful genotypes) in our study are remarkable and confirm the present multiplex SNP typing as a robust method. The analysis of the human Y chromosome has some technical advantages compared to other genomic regions, which could explain the high pass and call rates. Despite the complex and highly repetitive structure of the human Y chromosome [6], only one allele for each marker per individual is expected because of its uniparental inheritance and the choice of markers originating from unique regions. All male samples are thus hemizygous (presenting only one of the two alleles), and no heterozygous results are expected (or observed). This simplifies the calling, since only two clusters of results are expected (excluding the cluster of negative controls), one for each allele. Other genomic regions might be more difficult to genotype with the present technique, since the call rate must distinguish homozygous from heterozygous alleles.

The amount of DNA used in the present sample set as recommended by the manufacturer is high (300 ng); however, high pass rates and calling rates (similar to the ones provided in the present sample set) have been obtained with as little as 90 ng (thus a final amount of 45 ng used for genotyping), provided that the A260/A280 DNA absorbance ratio is approximately 1.8 (data not shown), which stresses the relevance of the DNA purity to perform the analyses. This DNA amount is within the range described in previous works multiplexing the Y chromosome, mitochondrial DNA and autosomal SNPs [10,12,16], although it is higher than that in other studies [11,13,14]. Nonetheless, this amount of DNA remains lower than the amount needed for genome-wide DNA chips (for example, 500 ng for the Genome-Wide Human SNP Array 6.0; Affymetrix, Inc., Santa Clara, CA 95051 USA; and 37.5 μg for the next-generation Omni microarrays derived from genome-wide association studies; Illumina, Inc. (San Diego, CA 92121-1975 USA), although these cover a much larger number of markers. In addition, the amount of DNA used in this study should not be a problem for population genetic studies, as the mean amount of human DNA extracted from 1 mL of saliva has been reported to be as much as 11.4 μg/mL [20] and even larger amounts are extracted from blood. The present assay might not be useful for forensic studies with very low amounts of DNA available (for example, extraction from a single hair or from degraded samples), although it can certainly be useful for forensic cases without a limited quantity of DNA (for example, paternity and family reunification analyses).

Although the present multiplex was designed to screen populations of European origin, it might be used to detect the presence of individuals of non-European ancestry in European samples and as a first approach for other admixed populations with subsequent typing for those haplogroups not common in Europe, since some branches of the Y-chromosome phylogeny are not defined in the present assay (that is, branches A or B or subbranches within C, D, K, M, N, O, P, Q or T). Nonetheless, the present results show that the TaqMan assay multiplex technology exhibits successful and robust results and that other combination of SNPs could be designed to genotype all branches of the Y-chromosome phylogeny or to focus on specific regions of the phylogeny. The OpenArray technology has the potential to combine up to 256 TaqMan assays in the same plate, which is enough to genotype the main diagnostic SNPs of most branches and subbranches of the human Y-chromosome phylogeny. In addition, the present methodology is flexible enough to include those markers on the Y chromosome that are required for a specific analysis which contrast with the rigidity of commercial high-throughput arrays that also include several SNPs in the Y chromosome. Although several genome-wide human SNP arrays have incorporated hundreds of Y-chromosome SNPs, just a few of the SNPs in our array share commonality with some commercially available SNP arrays (for instance, M216, M9 and P123 are included in the Genome-Wide Human SNP Array 6.0 manufactured by Affymetrix, Inc.; and M145 and M173 are included in the BeadArray Reader manufactured by Illumina, Inc.). Thus, to our knowledge, this study is the first to describe typing with flexibility to such a high accuracy the human Y chromosome in a single array.

## Conclusions

We have created and evaluated a robust and accurate Y-chromosome array to genotype 121 SNPs at a time using TaqMan probes that classifies individuals into the main haplogroups and the main European subhaplogroups in the human Y-chromosome phylogeny, substantially decreasing time of laboratory work and minimising the possible errors due to mixup when typing the same sample in several independent reactions.

## Methods

### Samples

DNA samples were obtained from 22 healthy unrelated males from each of the following 12 populations: Bulgarians, Bulgarian Roma, Spanish, Spanish Roma, Italians, Germans, British, Faroe Islanders, Danish, Greeks, Hungarians and Ukrainians. DNA samples from another 18 individuals of diverse origins obtained from the Coriell Institute for Medical Research (three from Taiwan, one Mbuti Pygmy, seven Russians, two Basques, one Chinese, two from the Middle East and two from Southeast Asia) were used as internal controls of the accuracy of the multiplex, since their haplogroup affiliations had previously been determined by two different laboratories using the same SNPs in single reactions incorporated into the present multiplex assay. Informed consent was obtained from all participants, and the project was approved by the Clinical Research Ethics Committee at the Institut Municipal d'Assistència Sanitària (IMAS reference 2006/2600/I). DNA extraction was carried out using a standard phenol/chloroform method.

### SNP selection

A total of 128 Y-chromosome SNPs defining the main haplogroups and subhaplogroups were chosen, with special attention to SNPs defining haplogroups and subhaplogroups reported as variable in European samples [17-19]. Data published by Karafet *et al*. [8] and four SNPs (P311, P312, L21, and L23) published on the Internal Society of Genetic Genealogy (ISOGG) 2009 Y-DNA SNP Index website (http://isogg.org/tree/ISOGG_YDNA_SNP_Index09.html) were used for SNP selection. The flanking sequences for each of the SNPs were then investigated for the design of the SNP TaqMan probes and submitted to the TaqMan assay design pipeline (Applied Biosystems, Inc.). See Additional file 2, Table S2, for a complete description of the primers used. Two different control assays were included in the multiplex. Two redundant assays (M145 and M203) for haplogroup DE, that is, SNPs that define the same Y-chromosome branch, were included as an internal phylogenetic control. In addition, two different assays for the same SNP (M9) were designed as an additional internal control. An M9a assay was designed to present the ancestral allele (C) in the probe with the VIC dye and the derived allele (G) with the FAM dye. The M9b assay was designed to present the ancestral allele (C) in the probe with the FAM dye and the derived allele (G) with the VIC dye.

### SNP genotyping and haplogroup classification

In the present analysis, a multiplex of 128 TaqMan assays was performed in a single array for each DNA sample. Each TaqMan OpenArray plate was designed to contain the 128 assays for a total of 24 samples (22 DNA samples and 2 NTCs). Technical exigencies of the autoloader machine require double the amount of DNA to be present in the loading tips. A total of 300 ng of genomic DNA was used, and a final amount of 150 ng was incorporated into the array with the autoloader and genotyped according to the manufacturer's recommendations. The multiplex TaqMan assay reactions were carried out in a dual 384-well GeneAmp 9700 Thermal Cycler (Applied Biosystems, Inc.) with the following PCR cycle: an initial step at 93°C for 10 minutes followed by 55 cycles of 45 seconds at 95°C, 13 seconds at 94°C and 2 minutes, 14 seconds at 53°C.

The fluorescence results were read using Autocaller software (Applied Biosystems, Inc.). The genotypes were compiled and used to assign each sample to its Y-chromosome haplogroup according to the Y-chromosome phylogeny published by Karafet *et al*. [8] and the Internal Society of Genetic Genealogy (ISOGG) 2009 Y-DNA SNP Index website (http://isogg.org/tree/ISOGG_YDNA_SNP_Index09.html).

## Competing interests

The authors declare that they have no competing interests.

## Authors' contributions

BMC and DC planned the study and wrote the manuscript. BMC analysed the data. JZ designed the assays. PS, GS, RA and SP performed the laboratory work. All authors read and approved the final manuscript.

## Supplementary Material

Additional file 1 **Figure S1**. Phylogenetic tree showing the haplogroup tested with the OpenArray following the nomenclature published by Karafet *et al*. [8] and on the Internal Society of Genetic Genealogy (ISOGG) 2009 Y-DNA SNP Index website (http://isogg.org/tree/ISOGG_YDNA_SNP_Index09.html). Recurrent mutations of three markers (SRY10831, P37 and P41) are shown in different branches of the tree. Click here for file

Additional file 2 **Table S2**. List of the primers used for the amplification of every assay implemented in the OpenArray. Click here for file
