# Supplementary figures and images for: Multiplex single-nucleotide polymorphism typing of the human Y chromosome using TaqMan probes
Source: Investig Genet. 2011 May 31;2:13. doi: 10.1186/2041-2223-2-13 (PMC3130649; doi:10.1186/2041-2223-2-13)

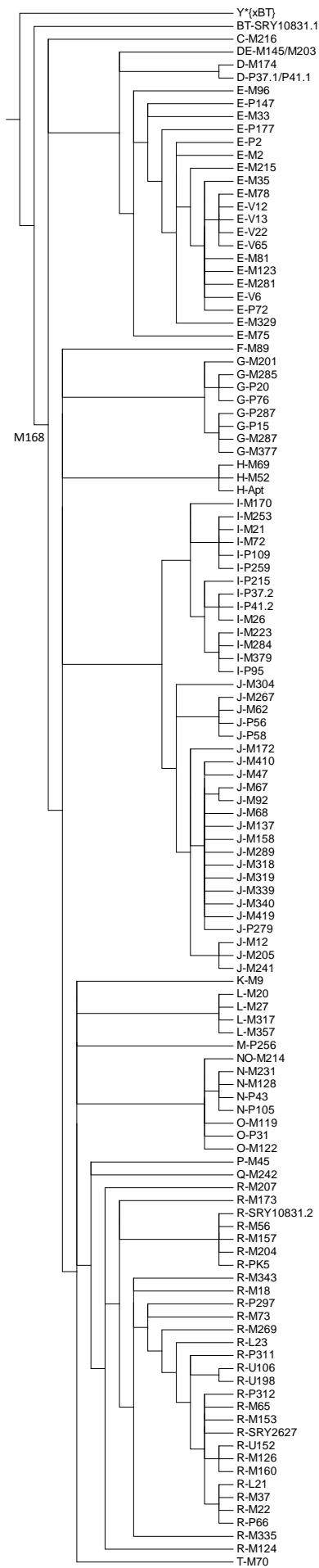

Supplement: Additional file 1 — Figure S1. Phylogenetic tree showing the haplogroup tested with the OpenArray following the nomenclature published by Karafet et al. [8] and on the Internal Society of Genetic Genealogy (ISOGG) 2009 Y-DNA SNP Index website (http://isogg.org/tree/ISOGG_YDNA_SNP_Index09.html). Recurrent mutations of three markers (SRY10831, P37 and P41) are shown in different branches of the tree. [file 2041-2223-2-13-S1.PDF]
